# Esthetic satisfaction vs. peri-implant health: a cautionary perspective

**DOI:** 10.2340/aos.v84.44055

**Published:** 2025-06-25

**Authors:** Mohammad Majduddin Sulaiman, Adam Husein, Ikhwan Hakimi Mohamad

**Affiliations:** a Prosthodontic Unit, School of Dental Sciences, Universiti Sains Malaysia, Kelantan, Malaysia; b Department of Restorative Dentistry, College of Dental Medicine, University of Sharjah, Sharjah, United Arab Emirates; c Periodontics Unit, School of Dental Sciences, Universiti Sains Malaysia, Kelantan, Malaysia

Dear Editor,

We read with great interest the recent article by Bengtsson et al. titled ‘Patient-reported outcomes of esthetics, function and oral hygiene with single dental implants 10–15 years after placement: a cross-sectional study’. The authors are to be commended for undertaking a long-term evaluation of single-implant-supported restorations in the esthetic zone, combining both patient-reported outcome measures (PROMs) and objective clinical indices such as pink esthetic score/white esthetic score (PES/WES). This dual approach reflects a much-needed emphasis on patient-centered care in implant dentistry.

However, we wish to raise several points that may contribute to a broader understanding and future development in this research area. First, while the authors acknowledged the lack of validated PROMs for esthetic assessment, this limitation significantly affects the reproducibility and generalizability of the findings. Given the increasing integration of PROMs in clinical trials and guideline development, the absence of a standardized, psychometrically sound instrument limits the interpretability of the high satisfaction scores reported [1]. This highlights an urgent need for the dental research community to develop and validate implant-specific PROMs that consider esthetic, functional, and psychological domains [2].

Second, the reported 88.9% prevalence of peri-implant mucositis is concerning, especially given the high patient satisfaction with esthetics and function. This highlights a potential disconnect between clinical findings and patient perception, suggesting that patients may be unaware of underlying peri-implant tissue inflammation, particularly when esthetic outcomes appear favorable. Notably, only one patient in the study reported regular visits to a dental hygienist, despite strong evidence that structured maintenance significantly reduces the risk of peri-implant disease. Non-adherence to maintenance care has been correlated with a higher incidence of peri-implant bone loss [3], and regular maintenance care has been shown to lower both the incidence and severity of peri-implant mucositis and peri-implantitis [4]. These findings underscore the critical role of long-term professional monitoring and patient education in ensuring peri-implant health.

Lastly, the study’s findings that PES scores were consistently lower than WES scores mirror trends observed in prior literature and reinforce the ongoing challenge in achieving optimal peri-implant soft tissue outcomes. However, the influence of initial tissue biotype, implant positioning protocol, and site-specific anatomical variations were not deeply analysed. Future studies incorporating 3D imaging, standardized mucosal thickness assessments, and patient satisfaction correlations could help refine guidelines for implant placement and restoration in the esthetic zone.

In conclusion, this study provides meaningful long-term insights and reinforces the need for holistic evaluations in implant dentistry. We congratulate the authors on their valuable contribution and hope this letter stimulates further discourse on optimizing both objective and subjective measures in implant success.
